# Individual, firearm, and purchasing characteristics associated with risk of firearm-related violent crime arrest: a nested case-control study

**DOI:** 10.1186/s40621-024-00534-0

**Published:** 2024-09-03

**Authors:** Hannah S. Laqueur, Julia P. Schleimer, Aaron B. Shev, Rose Kagawa

**Affiliations:** 1 Violence Prevention Research Program, Department of Emergency Medicine, University of California, Davis, Sacramento, CA USA; 2 California Firearm Violence Research Center, University of California, Davis, 2315 Stockton Blvd., Sacramento, CA 95817 USA

## Abstract

**Background:**

Firearm purchasing records offer a potentially important administrative data source to identify individuals at elevated risk of perpetrating firearm violence. In this study, we describe individual, firearm, and transaction characteristics of purchasers in California who were arrested for a firearm-related violent crime (FRV) as compared to the general population of registered purchasers in the state.

**Methods:**

Relying on a dataset of all individuals with transaction records in California (1996–2021), linked to criminal records (1980–2021), we enrolled a cohort of individuals for whom we could capture the *legal* firearm purchase history. We identified those arrested for FRV post purchase, and using incidence density sampling, gender-matched cases to ten purchasers (controls) who remained “at risk” at the time the case was arrested. We focused on the purchase closest in time prior to the arrest (“index” purchase). We implemented conditional logistic regression and included models with controls for individual- and community-level demographics, as well as interactions between firearm and purchasing characteristics and criminal history.

**Results:**

The cohort included 1,212,144 individuals, of whom 6153 were arrested for FRV (0.5%). Cases were matched to 61,530 controls to form the study sample. The largest risk factor was a prior criminal history: purchasers had 5.84 times the risk of FRV if they had a prior arrest within three years of the index purchase (CI 5.44–6.27). Several transaction and firearm characteristics were also associated with FRV. For example, risk increased if the firearm was redeemed at a pawn shop (aIRR: 1.37, CI 1.05–1.77) and decreased if the transaction was a registered private party transfer (vs. retail purchase) (aIRR: 0.83, CI 0.76–0.90) or the firearm was a bolt action firearm (vs. semi-automatic) (aIRR: 0.64, CI 0.51–0.79). In the interaction models, most of the purchase and firearm features only remained significant among those with no criminal history.

**Conclusions:**

Given limited data on firearm transactions, there has been little research on whether the type of firearm an individual purchases or the nature of the purchase might serve as indicators of risk for FRV. We found several transaction and firearm features were associated with risk of FRV. Notably, these features provided little evidence of additional risk for those with a prior criminal record.

**Supplementary Information:**

The online version contains supplementary material available at 10.1186/s40621-024-00534-0.

## Introduction

There are a number of well-established individual-level risk factors for firearm violence, including a history of violence, criminal arrest, substance use, and easy access to guns (Bjelopera et al. 2013). While the majority of criminal offenders obtain their firearms through social connections or the illicit market (Cook and Pollack 2017), many of the risk factors for firearm violence that have been documented in the general population have also been shown to apply to individuals who have passed a background check and legally acquired their firearms (Wintemute et al. 1998). The point of purchase represents an important intervention point, with both federal and state laws targeting and restricting individuals deemed high risk from purchasing a firearm; for example, in California, individuals with a violent misdemeanor conviction are prohibited from purchasing or possessing a firearm. Several longitudinal studies analyzing a cohort of individuals who legally purchased a handgun in California in 2001 found that those with prior convictions for alcohol-related offenses, which are non-prohibiting, had 2–3 times the risk of arrest for a subsequent violent crime (Laqueur et al. 2019; Kagawa et al. 2020); those with a prior simple assault arrest had 4 times the risk (Shev et al. 2023).

Among those with a criminal history, there is also research that suggests that acquiring a firearm increases risk. A study of individuals in California with violent misdemeanor convictions who legally purchased a handgun found that these individuals were at increased risk for subsequent crimes involving firearms or violence as compared to individuals with a similar criminal background who were denied purchase following the state’s 1991 law prohibiting those with violent misdemeanor convictions from purchasing firearms (Wintemute et al. 2001).

In addition to these individual-level risk factors, there is evidence from the literature on firearms recovered by law enforcement (so-called “crime guns”) that suggests that the type of firearm an individual purchases and the type of firearm sale may be associated with increased criminal risk. Firearms recovered in crimes are more likely to be inexpensive, semi-automatic, medium or larger caliber, short-barreled, and to have larger ammunition capacity (Koper 2007, 2013; Pierce et al. 2003; Wintemute et al. 2005; Wright et al. 2010). The crime gun literature has not, however, examined the relationship between these firearm and transaction features and individual purchaser risk of criminal arrest.

Finally, there is evidence from the literature on recovered crime guns, as well as research on mass shooters, that suggests firearm purchasing patterns may be important indicators of risk. Crime gun studies have found multiple purchases on the same day or over a short period of time (in states where this is possible) are positively associated with law enforcement recovery (Wright et al. 2010). Studies of mass shooters, though not our primary focus, indicate most mass shooters acquire their firearms through legal markets (Fox and DeLateur 2014; Follman et al. 2018), and often stockpile many firearms in close proximity to the attack (Schuurman et al. 2017). The identification and documentation of such patterns can be used to inform practice and prevention.

In this nested case-control study, we analyze more than two decades of legal firearm transaction records among purchasers in California. We examine whether firearm and transaction characteristics are associated with risk of arrest for firearm-related violent crime (FRV), and how these features interact with individual criminal history.

## Methods

### Study population

Our study population was drawn from a cohort of individuals who legally purchased a firearm in California from 1996 through October 2021. Data on purchasers and their firearm transactions were obtained from the California Department of Justice’s (CA DOJ) Automated Firearms System (AFS) database of Dealer Records of Sale (DROS). In California, essentially all firearm purchases or transfers in the state, including transfers between private parties, gifts, and loans, must be done through a Federal Firearm Licensee (FFL), who must send the records of sale or transfer to the CA DOJ where they are stored permanently in AFS. Additionally, individuals who move to California from out of state must report and register any firearms in their possession within 60 days of arrival. These records of sales, registrations, and transfers, have been recorded and maintained for all legal handgun transactions in the state since 1996; data on long guns have been stored since 2014. The records include the purchaser’s name, date of birth, sex, self-identified race and ethnicity, home address, and the date and time of the transaction.

We began by defining the cohort from which cases and controls were selected. All cohort members had a record of transaction in California during the period 1996–2021. To capture the full potential legal firearm purchasing records for individuals from the age at which they were legally eligible to purchase a firearm, we enrolled individuals based on age, over a twenty-five-year period (1996–2021): those with a record of purchase who were age 18 in 1996, aged 18–19 in 1997, and so on, up to individuals aged 18–43 in 2021. In California, until 2020, the legal age to purchase a long gun was age 18; the legal age to purchase a handgun is 21. This enrollment approach sacrifices the study of older purchasers. However, given our focus on interpersonal violence and the well-established finding that criminal risk peaks in the in late adolescents and early adulthood and declines significantly with age (Rocque et al. 2015), our primary interest was in younger individuals.

Importantly, we did not enroll or match on age at time of entry into the study population. That is, an individual who was 18 in 1996 could have, for example, purchased their first firearm in 2006 and entered the cohort at age 28. Follow-up time begins from the first purchase, and individuals are considered at risk until their first arrest for a firearm-related violent crime (FRV) or December 31, 2021.

### Outcome

Our outcome of interest was arrest for a violent offense (felony or misdemeanor) with a firearm. The CA DOJ provided criminal history records for individuals with a record of transaction in DROS. These records, stored in the Automated Criminal History System (ACHS), include all adult criminal history events in the state since 1980. Using offense codes and the level of the charge, crimes were determined to be violent using definitions set by the World Health Organization (2020). This included all major violent crimes such as rape, robbery, homicide, aggravated assault as well as simple assault crimes. Firearm use was determined from the specific offense codes, disposition of the offense, level of the offense, modifiers to an offense, and additional comments provided in the Record of Arrest and Prosecution (“RAP”) sheets. Details on the violent crime categorization and firearm-related offense codes are provided in the Supplemental Material (Table s3).

### Independent variables

Our key independent variables of interest related to the purchaser, their firearm transactions, and the firearm(s) themselves. Transaction characteristics included whether the transaction record was a pawn redemption, a registered private party transfer, voluntary registration (e.g. someone from out of state), non-roster peace officer transaction, or ‘other’ type of transaction, which included curio/relic registration and unique serial number application, as compared to a standard retail sale; and an indicator variable for whether the firearm was purchased at a retail store as compared to a gun show. Firearm characteristics included firearm category (revolver, semiautomatic pistol, or other), firearm type (handgun versus rifle or shot gun), caliber (categorized as small, e.g. 0.22, 0.25, 0.32; medium, e.g. 0.38, 0.3, 9 mm; and large, e.g. 0.40, 0.44, 0.45); and an indicator for whether the gun was “inexpensive,” which we proxied by the manufacturer, generating a possibly gapped histogram (Fushing and Roy 2018) of prices listed in the *Blue Book of Gun Values* (Fjestad and Beuning 2017). This data-driven histogram approach uses hierarchical clustering to determine cut-points such that the data is approximately uniform within each bin. We identified those manufactures in the lowest bin of prices.

Our primary interest was in the firearm purchase most proximal to the criminal event. Thus, if a purchaser had multiple purchases during the exposure period, we focused on the characteristics of the transaction and firearm pertaining to the purchase closest in time prior to the arrest (the “index” purchase). For the controls, this was the most recent purchase prior to selection as a control. We also included a count of the total number of transactions, if any, that an individual had made prior to the index purchase.

Individual-level variables included purchaser criminal history prior to the index purchase, coded as no prior arrest, an arrest within three years or less, or any arrests that were more than three years before the index purchase. We used this categorical coding to capture the fact that criminal arrest risk is highest after a criminal event and declines thereafter (Blumstein and Nakamura 2009; Kurlychek et al. 2007). In a sensitivity analysis, we coded these as any arrest within two years or less and arrests that were more than two years from the index purchase (Supplemental Material, Tables s5 and s6).

We included several purchaser demographic covariates that have been well-documented to be associated with the risk of arrest (Rocque et al. 2015; Bennett et al. 2005; Kagawa et al. 2021), and that we hypothesized might be associated with purchasing patterns. These included race and ethnicity (Asian, Black, Latinx, White, and other, which includes unknown or missing race and ethnicity), gender, and age at first purchase, derived from the driver’s license or ID provided at purchase. In a sensitivity analysis, we conducted a complete case analysis that excluded observations with unknown or missing race and ethnicity (2.6%).

Using the home address recorded in DROS, we geocoded purchaser addresses and identified the purchaser census tract to also adjust for community characteristics. We generated and included the first three principal components of socio-economic disadvantage measures comprised of: the Index of Concentration at the Extremes (ICE) for income, the proportion renters, proportion single parent households with children, proportion with bachelor’s degree or higher, proportion unemployed, median household income, median home value, and proportion receiving welfare. We also included ICE for race (considering Black residents as socially disadvantaged and White residents as socially privileged). Finally, we included rural–urban status (metro versus non-metro based on the USDA’s Rural–Urban Continuum Codes). All community characteristics pertained to the purchase address provided for the index purchase.

### Sampling

For each case, we used incidence density sampling to select ten controls from our pre-defined cohort who were still at-risk at the time of the case’s arrest. Under this sampling approach, controls may be randomly selected as controls more than once, and a person selected as a control may later become a case (Rothman 2008). Cases and controls were matched on gender for statistical efficiency.

### Statistical analysis

We analyzed the data using conditional logistic regression. This is mathematically identical to a stratified Cox model. Cases and controls are matched on time and gender and compared within risk sets using only data documenting past purchases and arrests as of the time of risk set sampling. With this sampling design, regression estimates should approximate incidence rate ratios (IRR) obtained from a cohort study (Vandenbroucke and Pearce 2012; Labrecque et al. 2021). We report multivariable adjusted incidence rate ratios (aIRR) and 95% confidence intervals (CI) with robust standard errors for the independent variables described above.

To capture the relationship between purchasing records and criminal history, we implemented a model in which we interacted purchase and firearm characteristics with the indicators for pre-index purchase criminal history within three years of purchase and more than three years prior to index purchase. Finally, we also ran a secondary analysis examining the relationship between any non-violent, non-firearm arrest post purchase and risk of FRV, controlling for all purchase features and individual and community characteristics (Supplemental Material, Table s2).

## Results

Table 1 presents descriptive characteristics of the study sample. Cases and controls differed significantly with respect to their prior criminal history. Among cases, 57% had an arrest prior to index purchase as compared to 21% of controls; 25% of cases had an arrest within three years or less of the index purchase (vs 6.4% of controls). Post purchase, arrests (for offenses other than the outcome) were also higher among cases. For example, 10% of cases had an arrest for a violent (non-firearm-related) crime (vs 2% of controls). Additional detail on the crime categories pre and post index arrests for cases and controls are provided in the Supplemental Material, Table s1. There were less dramatic differences between cases and controls with respect to the characteristics of their index purchase and purchasing history, but nonetheless, some differences. For example, among cases, the index purchase was more likely to be a low-cost firearm (5.4% vs 2.7%), the transaction was more likely to be a pawn redemption (1.3% vs 0.7%), and less likely to be a non-roster peace officer transaction (0.3% vs 1.3%).

**Table 1:** Description of study sample

Characteristics^a^	Case *N* = 6153	Control *N* = 61,530
*Purchaser characteristics*		
Age at index purchase	25.6 (22.7, 30.0)	26.8 (23.4, 31.2)
Unknown	1	0
Age at first purchase	24.4 (22.1, 28.3)	25.2 (22.5, 29.2)
Unknown	1	0
Gender		
Female	295 (5%)	2950 (5%)
Male	5858 (95%)	58,580 (95%)
Race and ethnicity		
American Indian	79 (1%)	427 (< 1%)
Asian	386 (6%)	5536 (9%)
Black	1036 (17%)	3360 (6%)
Hispanic	2129 (35%)	14,752 (24%)
Other	83 (1%)	738 (1%)
Pacific Islander	76 (1%)	596 (1%)
Unknown/missing	61 (1%)	933 (2%)
White	2303 (37%)	35,188 (57%)
*Purchaser community characteristics*		
ICE-income^b^	0.02 (− 0.13, 0.19)	0.11 (− 0.04, 0.30)
Proportion renters	0.38 (0.25, 0.54)	0.34 (0.21, 0.51)
Proportion single parent households with children	0.32 (0.24, 0.41)	0.28 (0.21, 0.36)
Proportion with bachelor’s degree or higher	0.16 (0.09, 0.26)	0.20 (0.12, 0.33)
Proportion overall unemployed	0.13 (0.09, 0.19)	0.11 (0.07, 0.16)
Median household income ($)	53,759 (41,055, 70,992)	62,013 (47,542, 81,735)
Median home value	206,755 (138,480, 302,470)	236,330 (160,645, 363,771)
Proportion receiving welfare	0.08 (0.04, 0.14)	0.05 (0.02, 0.10)
RUCA		
Metro	5139 (87%)	51,444 (86%)
Non-metro	771 (13%)	8644 (14%)
ICE-race^c^	0.23 (0.04, 0.51)	0.40 (0.15, 0.61)
Ratio of males to females aged 15–30 years	1.06 (0.99, 1.16)	1.08 (1.00, 1.17)
*Index purchase characteristics*		
Gun show purchase		
No	6045 (98%)	60,425 (98%)
Yes	107 (2%)	1105 (2%)
Transaction type		
Dealer retail sale	5123 (83%)	49,530 (80%)
Non-roster peace officer	16 (< 1%)	785 (1%)
Other	87 (1%)	1379 (2%)
Out of state registration	278 (5%)	2409 (4%)
Pawn redemption	80 (1%)	415 (< 1%)
Private party transfer	568 (9%)	7012 (11%)
Firearm type		
Handgun	4950 (80%)	47,247 (77%)
Rifle^d^	768 (12%)	9872 (16%)
Shotgun	434 (7%)	4406 (7%)
Firearm category		
4 or more barrels	0 (0%)	3 (< 1%)
Bolt action	106 (2%)	2204 (4%)
Carbine	4 (< 1%)	68 (< 1%)
Derringer	20 (< 1%)	122 (< 1%)
Double barrel	7 (< 1%)	67 (< 1%)
Lever action	25 (< 1%)	404 (< 1%)
Over and under	9 (< 1%)	181 (< 1%)
Pump action	351 (6%)	3276 (5%)
Revolver	423 (7%)	4618 (8%)
Semi-automatic	5,152 (84%)	50,027 (81%)
Single shot	52 (< 1%)	516 (< 1%)
Caliber		
Large	2285 (37%)	22,534 (37%)
Medium	2293 (37%)	21,705 (35%)
Other	1212 (20%)	14,379 (23%)
Small	362 (6%)	2,912 (5%)
Unknown	1 (< 1%)	0 (0%)
Low cost		
Yes	334 (5%)	1656 (3%)
No	5818 (95%)	59,874 (97%)
Total number prior purchases	1 (1, 2)	1 (1, 3)
*Pre-index purchase criminal arrests*		
Time from last pre-index purchase arrest to index purchase		
No pre-index purchase arrest	2648 (43%)	48,544 (79%)
Less than or equal to 3 years	1533(25%)	3956 (6%)
More than 3 years	1971 (32%)	9030 (15%)

^a^No. (%) and mean (1st, 3rd quartile)

^b^ICE-income ranges from − 1 (all residents belong to the least privileged group) to + 1 (all residents belong to the most privileged group), with least and most privileged groups classified as < $20 thousand and > $100 thousand annual income

^c^ICE-race ranges from − 1 (all residents belong to the least privileged group) to + 1 (all residents belong to the most privileged group), with least and most privileged groups classified as Black and White based on socially constructed hierarchies

Columns may not sum to total due to missing values

^d^Includes 27 rifle/shotgun combination firearms

Table 2 presents the results of the multivariate analysis of the association between index purchase characteristics and subsequent arrest for a FRV crime. Model 1 includes individual criminal history, model 2 includes criminal history and demographic and community controls. Both models condition by design on time and gender, the matching variables; analytically, this is implemented by using risk sets as strata in the model. As shown in model 1, compared to a standard retail sale, an individual whose index transaction was a pawn redemption had 1.48 times the risk of subsequent FRV crime arrest (CI 1.16–1.88). On the other hand, risk was significantly reduced if the transaction was a non-roster peace officer transfer (IRR: 0.29, CI 0.18–0.48), a registered private party transfer (IRR: 0.76, CI 0.70–0.83) or ‘other’ transaction type (IRR: 0.63, CI 0.51–0.79). Purchase of a low-cost firearm was also associated with increased risk for a subsequent FRV arrest (IRR: 1.58, CI 1.39–1.79). The purchase of a bolt action firearm, as compared to a semi-automatic, was associated with reduced risk (IRR: 0.61, CI 0.49, 0.75). The largest risk factor was prior criminal record: compared to purchasers with no criminal history, those with an arrest within three years prior to purchase had 6.82 times the risk of arrest for a FRV crime (CI 6.38–7.28) and four times the risk if their prior arrest was more than three years before the index purchase (IRR: 3.94, CI 3.71–4.17).

**Table 2:** Association between index purchase characteristics, prior criminal history, and subsequent arrest for firearm violent crime

	Model 1	Model 2
	OR (95% CI)	OR (95% CI)
*Index purchase characteristics*		
Gun show		
Yes	0.96 (0.79–1.16)	1.01 (0.84–1.23)
No	Ref	Ref
Transaction type		
Dealer’s sale	Ref	Ref
Non-roster peace officer	0.29 (0.18–0.48)	0.36 (0.21–0.60)
Other	0.63 (0.51–0.79)	0.73 (0.58–0.93)
Out of state registration	1.00 (0.89–1.14)	0.97 (0.81–1.18)
Pawn redemption	1.48 (1.16–1.88)	1.37 (1.05–1.77)
Private party transfer	0.76 (0.70–0.83)	0.83 (0.76–0.90)
Firearm type		
Handgun	Ref	Ref
Rifle^a^	0.66 (0.34–1.25)	0.70 (0.31–1.61)
Shotgun	0.55 (0.28–1.08)	0.73 (0.31–1.71)
Firearm category		
Bolt action	0.61 (0.49–0.75)	0.64 (0.51–0.79)
Other/unknown	1.08 (0.88–1.31)	1.07 (0.87–1.32)
Pump action	1.46 (1.16–1.83)	1.12 (0.88–1.43)
Revolver	0.91 (0.82–1.00)	0.97 (0.87–1.08)
Semi-automatic	Ref	Ref
Caliber		
Medium	0.95 (0.84–1.07)	0.87 (0.77–0.98)
Other	1.15 (0.60–2.20)	1.12 (0.49–2.57)
Small	Ref	Ref
Large	0.90 (0.80–1.02)	0.82 (0.73–0.93)
Low cost		
Yes	1.58 (1.39–1.79)	1.13 (0.99–1.30)
No	Ref	Ref
Total number prior purchases	1.00 (1.00–1.01)	1.02 (1.01–1.03)
Age at index purchase		0.94 (0.93–0.94)
*Criminal history*		
Time from last pre-index purchase arrest to index purchase		
No pre-index purchase arrest	Ref	Ref
Less than or equal to 3 years	6.82 (6.38–7.28)	5.84 (5.44–6.27)
More than 3 years	3.94 (3.71–4.17)	4.28 (4.01–4.56)
*Purchaser characteristics*		
Race and ethnicity		
American Indian		2.09 (1.66–2.63)
Asian		1.16 (1.03–1.30)
Black		2.93 (2.67–3.22)
Hispanic		1.59 (1.48–1.70)
Other		1.38 (1.10–1.73)
Pacific Islander		1.69 (1.32–2.15)
Unknown/missing		1.01 (0.72–1.42)
White		Ref
*Purchaser community characteristics*		
PC1^b^		1.11 (1.09–1.13)
PC2^b^		0.91 (0.88–0.94)
PC3^b^		0.93 (0.90–0.97)
RUCA		
Metro		Ref
Non-metro		0.99 (0.91–1.08)
ICE-race^c^		0.74 (0.65–0.84)
Ratio of males to females aged 15–30 years		0.93 (0.72–1.19)

Results from conditional logistic regression models with robust standard errors

^a^Includes rifle/shotgun combination firearms

^b^Principle components were created from ICE-income, proportion renters, proportion single parent households with children, proportion with bachelor’s degree or higher, proportion unemployed, median household income ($), median home value, and proportion receiving welfare. Three principles components explained 75% of the variance

^c^ICE-race ranges from − 1 (all residents belong to the least privileged group) to + 1 (all residents belong to the most privileged group), with least and most privileged groups classified as Black and White based on socially constructed hierarchies

These transaction, firearm, and criminal history features associated with FRV all remained significant in the model controlling for purchaser demographics and community characteristics (Table 2, model 2), apart from our proxy for a low-cost firearm purchase, which was no longer significantly associated with the outcome. Additionally, though caliber size was not significant in model 1, large caliber (relative to small) was statistically significant in a direction suggesting a protective association (aIRR: 0.83, CI 0.73–0.94) as was medium caliber (relative to small) (aIRR: 0.87, CI 0.77–0.98). Older age at index purchase was also associated with reduced risk: each additional year of age at index purchase was associated with 0.94 (CI 0.93–0.94) times the risk of subsequent FRV arrest. Several community characteristics were also statistically significant, with indicators of disadvantage associated with increased risk and less disadvantage associated with decreased risk.

In the supplementary model in which we include an indicator for a *post*-purchase arrest (other than the outcome), as well as pre-purchase arrests and all variables included in models 1 and 2, (Supplemental Material, Table s2), we find a post purchase (non-outcome) arrest was associated with roughly twice the risk of FRV arrest: IRR: 2.25, CI 2.09–2.34, excluding individual demographic and community controls, and aIRR: 1.93 (CI 1.78–2.09) in the model including these controls.

Finally, Table 3 presents the interactions between index firearm purchase characteristics and criminal history and their associations with subsequent arrest for a FRV crime. The transaction types, as well as the inexpensive make indicators that were significant in models 1 and 2, are significantly associated with risk of FRV among those individuals with *no* pre-index purchase arrest history, but not among purchasers with a criminal record. Among this group with no prior arrest history, the purchase of a low-cost handgun was associated with a 49% increase in risk (aIRR: 1.49, CI 1.23–1.80) and a pawn redemption was associated with a 73% increase in risk (aIRR: 1.73 (1.20–2.49).

**Table 3:** Interactions between index firearm purchase characteristics and criminal history in their association with subsequent arrest for firearm violent crime

	Time from last pre-index purchase arrest to index purchase		
	Among those with no arrest	Among those whose last pre-index purchase arrest was less than or equal to 3 years before index purchase	Among those whose last pre-index purchase arrest was more than 3 years before index purchase
	OR (95% CI)	OR (95% CI)	OR (95% CI)
*Low cost*			
Yes	1.49 (1.23–1.80)	0.96 (0.75, 1.22)	0.88 (0.69, 1.11)
No	Ref	Ref	Ref
*Transaction type*			
Dealer’s sale	Ref	Ref	Ref
Non-roster peace officer	0.43 (0.26–0.71)	–^a^	0.11 (0.01, 0.82)
Other	0.63 (0.46–0.88)	0.84 (0.45, 1.56)	0.89 (0.61, 1.31)
Out of state registration	0.86 (0.63–1.18)	1.05 (0.79, 1.38)	1.07 (0.78, 1.47)
Pawn redemption	1.73 (1.20–2.49)	0.94 (0.58, 1.51)	1.37 (0.87, 2.15)
Private party transfer	0.72 (0.62–0.83)	1.04 (0.87, 1.25)	0.86 (0.74, 1.01)
*Firearm type*			
Handgun	Ref	Ref	Ref
Rifle^b^	0.67 (0.29–1.55)	0.96 (0.41, 2.24)	0.66 (0.29, 1.55)
Shotgun	0.69 (0.29–1.63)	1.05 (0.43, 2.53)	0.65 (0.27, 1.54)
*Caliber*			
Medium	0.91 (0.76–1.09)	0.86 (0.67, 1.1)	0.82 (0.67, 1.01)
Other	1.16 (0.50–2.71)	1.60 (0.67, 3.82)	0.97 (0.41, 2.28)
Small	Ref	Ref	Ref
Large	0.89 (0.74–1.07)	0.84 (0.65, 1.08)	0.73 (0.60, 0.90)

Results from conditional logistic regression models. All models control for gun show, transaction type, firearm type, caliber, cost, number of purchases, and purchaser demographic and community covariates as in Model 2, Table 2. Significant interactions at alpha 0.20 (likelihood ratio test) are presented

^a^Estimate not presented due to sparse data

^b^Includes rifle/shotgun combination firearms

All of our results were comparable in the sensitivity analyses (Supplement Material, Tables s4–s7).

## Discussion

We found several features related to the firearm transaction and the firearm itself to be significantly associated with subsequent risk of FRV arrest, though these associations were of much smaller magnitude than the risk associated with prior criminal arrest. For example, controlling for criminal history, individual demographic characteristics and community characteristics, a legally conducted private party transfer, compared to a standard retail sale, was associated with a 17% reduction in risk of arrest. That a registered private party transfer was associated with reduced risk is perhaps not surprising as it represents a transaction involving individuals who went out of their way to abide the law in conducting and undergoing a background check and recording their private party transaction. Not represented in our data are the individuals who acquired a firearm via private party transfer without conducting a background check and recording the sale. Estimates from survey research suggests that in comprehensive background check states approximately 40% of individuals who purchased a firearm from a private party did not undergo a background check (75% in states without comprehensive background check laws) (Hepburn et al. 2022). Nationally, unlicensed firearm dealing by a private party is the most common source by which firearms are trafficked (Bureau of Alcohol, Tobacco, Firearms and Explosives). The reduced risk we find associated with registered private party transfers could reflect effectiveness of laws such as California’s, which requires private party transfers to be conducted with both parties, in person, through a fully licensed firearms dealer. Alternatively, it may simply reflect the fact that individuals who follow the law in a context in which many individuals don’t, are particularly law abiding. We also found that transactions categorized as ‘other,’ which includes the registration of a collector’s item or the registration of a unique serial number registration, were associated with a 27% reduction in risk. This similarly reflects, in part, purchasers who have gone out of their way to obey the law in obtaining a serial number; this was a law implemented to combat the rise of privately manufactured firearms (so-called “ghost guns”), which, without a serial number, are untraceable and much more often used in crimes (Office of the Attorney General 2024; Braga et al. 2022).

On the other hand, compared to a standard retail sale, a pawn redemption was associated with an estimated 37% increase in risk. This is consistent with crime gun research, which has found that handguns acquired from pawn shops are more likely to later be recovered in crimes by law enforcement (Koper 2014; Robinson et al. 2024). Recovered handguns are also more likely to be inexpensive (Wintemute 2009), and we find that an individual’s purchase of an inexpensive firearm (by our proxy measure) was associated with increased risk of later arrest for FRV (IRR: 1.58, CI 1.39–1.79). However, when we include the additional control variables (model 2), the association was no longer statistically significant. Finally, though research suggests that firearms purchased at gun shows may be more likely to be used in crime (Braga 2017), and gun shows have been a target of the Bureau of Alcohol, Tabacco and Firearms and Explosives (ATF) investigations, (Krouse 2010) we do not find a significant association between an individual making a purchase at a gun show and FRV risk. This is in line with previous research from California showing a short-term increase in gun violence following gun shows in Nevada, a state with few regulations, but no association between California gun shows and local gun violence (Matthay et al. 2017). This absence of an association between gun shows in California and gun violence may be due to the presence of comprehensive gun show regulations and oversight in the state. In many other states, gun shows are not subject to the same federal regulations as are licensed firearms dealers (the so-called “gun show loophole”) (Gobaud et al. 2022), and so are more likely to involve unlicensed vendors and private parties who are not required to initiate a background check or maintain records of the purchase. Finally, as with private party transactions more generally, we do not have records for any transactions at gun shows that might have been conducted off-the-books and so we do not have records for these higher risk transfers.

While we document several important features related to the transaction and the firearm type purchased, a prior criminal history was the most important risk factor considered. This is consistent with the well-established finding that criminal history represents one of the strongest predictors for future criminal arrest or conviction (Council 2013) and risk of new criminal arrest is highest shortly after a recent criminal event and declines thereafter (Blumstein and Nakamura 2009; Kurlychek et al. 2007). Purchasers with a criminal arrest within the three years prior to the index firearm purchase had close to six times the risk of being arrested for a FRV offense compared to those with no criminal history, and those with an arrest more than three years prior had more than four times the risk. A criminal arrest for a non-firearm or non-violent offense following the index purchase was also associated with an increased risk for FRV, representing a second potential point of intervention. A prior criminal record was also relatively common: 57% of cases and 21% of controls had at least one arrest prior to their index purchase. Thus, the magnitude of the association for this risk factor was not only larger than were the associations with purchase and firearm characteristics, but it was also more prevalent in the sample of purchasers.

The association between criminal record and subsequent FRV arrest among individuals who passed a background check and legally purchased a firearm in California is in line with previous research using a cohort of individuals in California who legally purchased a firearm in 2001. These studies found associations with subsequent risk for a violent charge across multiple criminal history charge types (Laqueur et al. 2019; Kagawa et al. 2020; Shev et al. 2023). It is worth noting that the associations observed in California-based studies are not generalizable to other states due to California’s unique and particularly stringent set of firearm purchase disqualifiers. In particular, people convicted of violent misdemeanors are not eligible to legally purchase a firearm. As a result, the magnitude of the associations with criminal history observed in this study, like in previous California-based studies, is likely smaller than it would be in a state without violent misdemeanor prohibitions.

A few of our findings were somewhat counter-intuitive. For example, although the crime gun literature has shown large and medium caliber handguns are more often recovered in crime (Koper 2014; Robinson et al. 2024), in the model controlling for individual and community characteristics (model 2), we found large and medium caliber size (relative to small) were associated with lower individual risk of subsequent arrest for FRV. The difference may be explained by the selection bias inherent in the crime gun literature focus on only those firearms used in crimes that are recovered; or it may reflect differences in the set of individual demographic and community controls.

Notably, most of the transaction and firearm characteristics that we found to be associated with a subsequent FRV arrest were only significant among purchasers without a criminal record; these factors seem to have little association with risk among those with prior criminal arrests. Thus, while criminal history remains the most important predictor, these purchase and transaction features do represent additional protective and risk indicators among those with no arrest history before their index purchase. At the same time, these purchasers, and our study population more generally, remain a low-risk group: only 0.5% of our study population was arrested for a firearm-related violent crime. Thus, even a doubling of risk is still a low overall risk of FVR.

## Limitations

There are several important limitations to note. First, this is necessarily a study of only legal purchasers and their legal purchases. These data are not a representative sample of firearm acquisitions or of perpetrators of gun-related violent crime. As noted in the introduction, most gun crimes are committed by individuals who did not legally obtain their firearm (Cook and Pollack 2017; Fabio et al. 2016). Not only are we missing illicit or underground market acquisitions and non-registered private party transfers, but our dataset of officially reported transactions and transfers is likely missing data on firearms owned by individuals moving to California from out-of-state, who may not know to register their firearms upon arrival or avoid doing so. Our study population of individuals who legally purchased or registered one or more firearms is not a high-risk population, and FVR among this group is rare. Finally, among those purchasers in our study who were arrested for FRV, we do not know if they acquired additional firearms illegally, and we do not know if the firearms for which we do have records were those that were used in the FRV crime for which the purchaser was arrested.

Our study sample is also not representative of all legal firearm purchasers in the state. We restricted enrollment based on age to better observe the complete history of potential individual firearm purchasing. We therefore have more dense observations for individuals in their twenties, and no observations for individuals over 43. Given our outcome of interest and the well-established fact that crime and violence peaks in late adolescents and early adulthood and declines significantly with age (Rocque et al. 2015; Farrington et al. 2012), this enrollment restriction that excludes older individuals is of less concern. Even with the age enrollment restriction, we do not capture purchases of long guns prior to 2014, as these data were not consistently collected in the CA DOJ Dealer Record of Sale (DROS) database. Here too, while a limitation, very few firearm-related violent crimes are perpetrated with rifles or shot guns (Federal Bureau of Investigation 2019).

It is also important to note that this is a study of criminal *arrests*, which only serve as an imperfect proxy for criminal behavior. Many crimes go unreported and undetected, and the degree of under ascertainment varies by crime type; for example, crimes of murder capture nearly all cases of the behavior whereas crimes of sexual abuse capture only a fraction of the behavior (Scurich 2020). Additionally, charges and arrests include some cases where the underlying behavior was in fact, not present (e.g. when there was the wrong suspect). Finally, criminal arrest and charge data also reflect differential police presence and police action in communities of color (Geller and Fagan 2010), particularly in the context of discretionary crimes such as drug possession or Driving Under the Influence (DUI) (Ketchum and Peck 2022). For example, Latino men are more like to receive a DUI conviction than are White or Asian American men with similar levels of engaging in alcohol-impaired driving (Kagawa et al. 2021). While the extent to which differential treatment (e.g. due to police patrolling of disadvantaged neighborhoods) versus differential involvement—stemming from longstanding systematic racism and disadvantage—explains racial differences in criminal arrests continues to be debated (Beck and Blumstein 2018), studies comparing crime victimization surveys with self-reported offending suggest that racial disparities in serious violent crime arrests are largely explained by differential rates of behavior rather than differential detection (Skeem and Lowenkamp 2016). Thus, our outcome, violent crime with a firearm, is likely less subject to bias. Nonetheless, criminal history as a risk factor may be differentially important across racial and ethnic groups.

## Conclusions

Given limited data on firearm transactions, there has been little research on whether the type of firearm an individual purchases or the nature of their purchase might serve as indicators of risk for subsequent firearm-related violent crime. California is the only state in which individual-level firearm transaction records are maintained and made available for research. In this study, we found several types of firearm transactions and several features of the firearm were associated with a purchaser’s risk of subsequent firearm-related violent arrest. Notably, these characteristics were largely only relevant among those who had no prior criminal record. Thus, we were able to identify risk and protective factors among a population that is overall lower risk. At the same time, these features provide little evidence of additional risk for those who have a prior criminal record, and the risk associated with a criminal record is of much larger magnitude than the risk and protective characteristics of the transaction or firearm.

## Supplementary Information

Additional file 1.

## Data Availability

The data analyzed in this study are from the California Department of Justice. The data use agreement does not permit data sharing.
