# Supplementary material for: Individual, firearm, and purchasing characteristics associated with risk of firearm-related violent crime arrest: a nested case-control study
Source: Inj Epidemiol. 2024 Sep 3;11:42. doi: 10.1186/s40621-024-00534-0 (PMC11373450; doi:10.1186/s40621-024-00534-0)
Supplement: Supplementary file 1 — Additional file 1. [file 40621_2024_534_MOESM1_ESM.docx]

**Supplementary Material**

Individual, firearm, and purchasing characteristics associated with risk of firearm-related violent crime arrest: a nested case-control study

Hannah S Laqueur,^a,b^ Julia P Schleimer, ^a,b^ Aaron B Shev, ^a,b^ Rose Kagawa ^a,b^

^a^Violence Prevention Research Program, Department of Emergency Medicine, University of California, Davis, USA

^b^California Firearm Violence Research Center, University of California, Davis, USA 2315 Stockton Blvd., Sacramento, CA, 95817, USA 

Table s1. Description of Study Sample, Detailed Criminal History

| *Criminal arrests by type pre-index purchase* |  |  |
| --- | --- | --- |
| **Major violent crime arrests** |  |  |
| 0 | 5,572 (91%) | 60,310 (98%) |
| 1+ | 581 (9%) | 1,220 (2%) |
| **Property arrests** |  |  |
| 0 | 4,877 (79%) | 58,327 (95%) |
| 1+ | 1,276 (21%) | 3,203 (5%) |
| **Alcohol arrests** |  |  |
| 0 | 4,516 (73%) | 55,439 (90%) |
| 1+ | 1,637 (27%) | 6,091 (10%) |
| **Any violent arrests** |  |  |
| 0 | 4,971 (81%) | 58,844 (96%) |
| 1+ | 1,182 (19%) | 2,686 (4%) |
| **Firearm violent arrests** |  |  |
| 0 | 6,096 (99%) | 61,394 (100%) |
| 1+ | 57 (<1%) | 136 (<1%) |
| **Firearm non-violent arrests** |  |  |
| 0 | 5,872 (95%) | 60,924 (99%) |
| 1+ | 281 (45%) | 606 (1%) |
| *Criminal arrests post-index purchase* |  |  |
| **Major violent crime arrests** |  |  |
| 0 | 5,766 (94%) | 60,713 (99%) |
| 1+ | 387 (6%) | 817 (1%) |
| **Property arrests** |  |  |
| 0 | 5,866 (95%) | 60,878 (99%) |
| 1+ | 287 (5%) | 652 (1%) |
| **Alcohol arrests** |  |  |
| 0 | 5,460 (89%) | 59,132 (96%) |
| 1+ | 693 (11%) | 2,398 (4%) |
| **Any violent arrests** |  |  |
| 0 | 5,562 (90%) | 60,272 (98%) |
| 1+ | 591 (10%) | 1,258 (2%) |
| **Firearm non-violent arrests** |  |  |
| 0 | 5,777 (94%) | 60,797 (99%) |
| 1+ | 376 (6%) | 733 (1%) |

Table s2. Association between post-index purchase criminal history and subsequent arrest for firearm violent crime

|  | **Model 1** | **Model 2** |
| --- | --- | --- |
|  | **OR (95% CI)** | **OR (95% CI)** |
| Any post-index purchase arrest |  |  |
| No | Ref. | Ref. |
| Yes | 2.25 (2.09-2.43) | 1.93 (1.78-2.09) |

Results from conditional logistic regression models with robust standard errors. Includes the same set of variables as the primary models including pre-index purchase arrests, firearm characteristics, transaction characteristics and individual and community control variables.

Table s3. Offenses Categorized as Violent

| CODE | SECTION | SUB-SECTION(S) | DESCRIPTION | UCR | FIREARM-RELATED VIOLENCE |
| --- | --- | --- | --- | --- | --- |
| P | 187 | (a) | First degree murder | 1 | x |
| P | 192 | (a) | Voluntary manslaughter | 1 |  |
| P | 261 | (a), (a)(1), (a)(2), (a)(3), (a)(4) | Rape by force or threat | 2 |  |
| P | 262 | (a) | Rape of spouse by force/threat/fear | 2 |  |
| P | 264.1 |  | Rape/etc. in concert with force/violence | 2 |  |
| P | 211^§^ |  | Robbery | 3 | x |
| P | 212.5 | (a), (b), (c)^§^ | Robbery of inhabited building, vehicle, ATM | 3 | x |
| P | 213.5 | (a)(1)(A), (a)(2) | Robbery of inhabited dwelling, in concert with others | 3 |  |
| P | 215 | (a)^§^ | Carjacking | 3 | x |
| P | 148 | (b) | Remove weapon from public officer - not firearm | 4 |  |
| P | 149 |  | Assault by public officer | 4 |  |
| P | 203 |  | Mayhem | 4 |  |
| P | 217 |  | Assault w/intent to kill | 4 |  |
| P | 220 |  | Assault with intent to commit rape | 4 |  |
| P | 221 |  | Assault to commit other felony | 4 |  |
| P | 240 |  | Assault | 4 |  |
| P | 241 | (a), (b), (c) | Assault peace officer | 4 |  |
| P | 241.1 |  | Assault on custodial officer | 4 |  |
| P | 241/243 |  | Assault and battery on peace officer /emergency personal | 4 |  |
| P | 243 | (a), (b), (c)(1), (c)(2), (d) | Battery on peace officer/emergency personal w/injury | 4 |  |
| P | 243.1 |  | Battery on custodial officer | 4 |  |
| P | 243.4 | (a), (b), (c), (d), (e)(1) | Sexual battery | 4 |  |
| P | 243.6 |  | Assault or battery on process server | 4 |  |
| P | 244 |  | Assault with caustic chemical/etc | 4 |  |
| P | 245 | (a), (a)(1), (a)(2), (a)(4), (b), (c) | Assault with deadly weapon or force: possible great bodily injury: not firearm | 4 |  |
| P | 245 | (a)(1), (a)(2), (a)(3), (a)(4), (b), (c), (d)(1), (d)(2), (d)(3) | Assault with deadly weapon: firearm | 4 | x |
| P | 246 |  | Shoot at inhabited dwelling/vehicle/etc. | 4 | x |
| P | 246.3 | (a), (b) | Willful discharge of firearm with gross negligence | 4 | x |
| P | 269(A)(1) | (a)(1), (a)(4) | Aggravated sexual assault of child/minor | 4 |  |
| P | 273.5 | (a) | Inflict corporal injury: spouse/cohabitee/date | 4 |  |
| P | 273.55 |  | Inflict corporal injury: spouse/cohabitee/date: special circumstances | 4 |  |
| P | 273a | (1), (a), (a)(1) | Willful cruelty to child: possible injury/death | 4 |  |
| P | 273d | (a) | Inflict injury upon child | 4 |  |
| P | 368 | (a), (a)(1),  (b)(1), (c) | Cruelty of elderly/dependent adult with great bodily injury/death or mental suffering | 4 |  |
| P | 405a |  | Lynching | 4 |  |
| P | 417 | (b), (c) | Exhibit firearm in presence peace officer | 4 | x |
| P | 417.1 |  | Exhibit firearm in presence of reserve police officer | 4 | x |
| P | 417.3 |  | Occupant of motor vehicle exhibit/draw firearm | 4 | x |
| P | 422 | (a)^§^ | Threaten crime with intent to terrorize | 4 | x |
| P | 422.7 | (a) | Violate civil rights by force/treat | 4 |  |
| P | 4501 |  | Assault by prisoner | 4 |  |
| P | 4501.5 |  | Battery by prisoner | 4 |  |
| P | 664/187 | (a) | Attempted murder | 4 |  |
| P | 69 |  | Obstruct/resist executive officer | 4 |  |
| P | 76 | (a) | Threaten/etc. elected official/judge/etc. | 4 |  |
| V | 23110 | (b) | Throw substance at vehicle with intent of great bodily injury | 4 |  |
| P | 136 | (b) | Prevent/dissuade witness/victim by force/etc. | 9 |  |
| P | 136.1 | (a)(2), (b)(1), (b)(2), (b)(3), (c), (c)(1) | Prevent/dissuade witness/victim by force/etc. | 9 |  |
| P | 140 | (a) | Threaten witness/victim of crime | 9 |  |
| P | 148 | (a), (a)(1) | Obstruct/resist public officer | 9 |  |
| P | 148.2.1 |  | Interfere with/etc. fireman/rescuer | 9 |  |
| P | 148.2.2 |  | Disobey order of fireman/public officer | 9 |  |
| P | 240^ǂ^ |  | Assault | 9 |  |
| P | 240/242 |  | Assault & battery | 9 |  |
| P | 241 | (a) ^ǂ^, (b) ^ǂ^ | Assault on peace officer/emergency personal | 9 |  |
| P | 241.4 |  | Assault school district peace office | 9 |  |
| P | 241.6 |  | Assault on school employee | 9 |  |
| P | 241/243^ǂ^ |  | Assault and battery on peace officer /emergency personal | 9 |  |
| P | 242 |  | Battery | 9 |  |
| P | 242/243 | (a), (b) | Battery on peace officer /emergency personal | 9 |  |
| P | 243 | (e)(1) | Bat Battery: spouse/ex-spouse/date/etc. | 9 |  |
| P | 243.2 | (a) | Battery on person on school property | 9 |  |
| P | 243.3 |  | Battery on transportation personnel | 9 |  |
| P | 243.4 | (a) ^ǂ^, (d) ^ǂ^, (d)(1) | Sexual battery | 9 |  |
| P | 243.5 | (a)(1) | Assault or battery on school property | 9 |  |
| P | 243.6^ǂ^ |  | Assault or battery on process server | 9 |  |
| P | 244.5 | (b) | Assault with stun gun/taser | 9 |  |
| P | 245 | (a)(1) ^ǂ^ | Assault w/deadly weapon: not firearm | 9 |  |
| P | 273a | (b) | Willful cruelty to child | 9 |  |
| P | 273d | (a) ^ǂ^ | Inflict injury/etc. upon child | 9 |  |
| P | 368 | (b), (c) ^ǂ^ | Cruelty to dependent adult | 9 |  |
| P | 69^ǂ^ |  | Obstruct/resist executive officer | 9 |  |
| P | 71 |  | Threaten school/public officer/employee | 9 |  |
| U | 18 113 | (e) | Simple assault | 9 |  |
| P | 146a |  | Impersonate public officer/etc. | 11 |  |
| P | 12024 |  | Possess deadly weapon: commit assault | 15 |  |
| P | 12034 | (b) | Discharge firearm from vehicle | 15 | x |
| P | 12303.2 |  | Possess explosive/etc. device in public | 15 |  |
| P | 12303.3 |  | Use/etc. explosive/etc. device: intent to injury | 15 |  |
| P | 12355 | (b) | Possess boobytrap device with intent to use | 15 |  |
| P | 12403.7 | (a), (a)(5), (a)(7), (a)(8) | Illegal possession/use of tear gas/tear gas weapon | 15 |  |
| P | 246.3 | (a), (b) | Willful discharge of firearm with gross negligence | 15 | x |
| P | 248 |  | Impair aircraft with light/etc. | 15 |  |
| P | 417 | (a), (a)(2) | Exhibit deadly weapon/firearm | 15 | x |
| P | 417 | (a)(1) | Brandishing weaponry, not firearm | 15 |  |
| P | 417.25 | (a) | Threaten person with laser scope with intention of causing fear | 15 |  |
| P | 417.4 |  | Brandishing firearm replica | 15 | x |
| P | 467 |  | Possess weapon to commit assault | 15 |  |
| P | 286 | (d)(1) | Sodomy in concert with force | 17 |  |
| P | 288 | (b)(1) | Lewd and lascivious acts with child under 14 yrs.: with force/etc. | 17 |  |
| P | 288.5 | (a) | Continuous sexual abuse of child | 17 |  |
| P | 288a | (a), (b), (d) | Oral copulation | 17 |  |
| P | 289 | (a), (a)(1), (b) | Sexual penetration with foreign object/etc. with force/etc. | 17 |  |
| P | 273a | (2) | Willful cruelty to child | 20 |  |
| P | 277 |  | Deprive custody right of another | 20 |  |
| P | 415 | (1), (2), (3) | Fight/challenge fight/ unreasonable noise/offensive words in public place | 24 |  |
| P | 415.5 | (a)(1) | Fight/challenge fight on university/etc. | 24 |  |
| U | 36 2.34 | (a)(1) | Disorderly conduct: fight/etc. | 24 |  |
| H | 12680 |  | Discharge fireworks: likely to injure | 26 |  |
| L | 6425 |  | Cause employee death/impairment | 26 |  |
| P | 136 |  | Prevent/dissuade witness/victim | 26 |  |
| P | 136.1 | (a), (a)(2), (b), (b)(1), (b)(2) | Prevent or dissuade witness from giving testimony | 26 |  |
| P | 146 | (a) | Make arrest/etc. without authority | 26 |  |
| P | 147 |  | Inhumanity to prisoners | 26 |  |
| P | 148.1 | (a), (b), (c), (d) | False bomb report | 26 |  |
| P | 148.3 | (a) | False report of emergency | 26 |  |
| P | 207 | (a), (b) | Kidnapping | 26 |  |
| P | 209 | (a), (b), (b)(1) | Kidnapping for ransom or to commit robbery/rape/etc. | 26 |  |
| P | 236^§^ |  | False imprisonment | 26 | x |
| P | 237 | (a) | False imprisonment with violence | 26 |  |
| P | 278 |  | Child stealing | 26 |  |
| P | 375 | (a) | Offensive/etc. matter in public place | 26 |  |
| P | 404.6 | (a) | Urge riot or destroy property | 26 |  |
| P | 405 |  | Riot | 26 |  |
| P | 422 | (a) ^ǂ^ | Threaten crime with intent to terrorize | 26 |  |
| P | 422.6 | (a) | Violate civil rights by force/threat | 26 |  |
| P | 4532 | (a), (b) | Escape jail/etc. with force/violence | 26 |  |
| P | 518 |  | Extortion | 26 |  |
| P | 519 |  | Extortion by threat | 26 |  |
| P | 520 |  | Extortion of property | 26 |  |
| P | 601 | (a)(1) | Trespass on residence: execute threat | 26 |  |
| V | 23110 | (a) | Throw substance at vehicle | V^€^ |  |
| V | 2800.2 |  | Evade peace officer in vehicle disregarding safety | V^€^ |  |
| V | 2800.3 |  | Evade peace officer: cause great bodily injury/death | V^€^ |  |

^a^ Code: H = California Health and Safety code; L = California Labor code; P = California Penal code; U = Code of Federal Regulations; V = California Vehicle code

x Categorized as gun offense if a firearm was used in the commission of the crime

€ Not a UCR code; categorized as a vehicle code violation.

**Sensitivity Analyses**

Table s4. Association between index purchase characteristics, prior criminal history, and subsequent arrest for firearm violent crime, *complete case analysis (n = 65,935)*

|  | **Model 1** | **Model 2** |
| --- | --- | --- |
|  | **OR (95% CI)** | **OR (95% CI)** |
| **Index purchase characteristics** |  |  |
| Gun show |  |  |
| Yes | 0.97 (0.80-1.18) | 1.01 (0.83-1.23) |
| No | Ref. | Ref. |
| Transaction type |  |  |
| Dealer’s sale | Ref. | Ref. |
| Non-roster peace officer | 0.30 (0.18-0.50) | 0.36 (0.22-0.60) |
| Other | 0.63 (0.50-0.79) | 0.73 (0.58-0.92) |
| Out of state registration | 0.97 (0.82-1.16) | 1.00 (0.83-1.21) |
| Pawn redemption | 1.51 (1.18-1.93) | 1.38 (1.07-1.79) |
| Private party transfer | 0.77 (0.70-0.84) | 0.83 (0.76-0.91) |
| Firearm type |  |  |
| Handgun | Ref. | Ref. |
| Rifle^a^ | 0.82 (0.34-1.94) | 0.69 (0.30-1.60) |
| Shotgun | 0.68 (0.28-1.65) | 0.70 (0.30-1.66) |
| Firearm category |  |  |
| Bolt action | 0.57 (0.46-0.71) | 0.63 (0.51-0.78) |
| Other | 1.02 (0.82-1.26) | 1.07 (0.87-1.31) |
| Pump action | 1.46 (1.15-1.86) | 1.15 (0.90-1.46) |
| Revolver | 0.92 (0.83-1.02) | 0.98 (0.88-1.09) |
| Semi-automatic | Ref. | Ref. |
| Caliber |  |  |
| Medium | 0.93 (0.82-1.05) | 0.86 (0.76-0.98) |
| Other | 0.93 (0.39-2.22) | 1.12 (0.48-2.58) |
| Small | Ref. | Ref. |
| Large | 0.89 (0.78-1.00) | 0.81 (0.72-0.92) |
| Low cost |  |  |
| Yes | 1.60 (1.41-1.82) | 1.13 (0.99-1.30) |
| No | Ref. | Ref. |
| Total number prior purchases | 1.00 (0.99-1.01) | 1.02 (1.01-1.03) |
| Age at index purchase |  | 0.94 (0.93-0.94) |
| **Criminal history** |  |  |
| Time from last pre-index purchase arrest to index purchase |  |  |
| No pre-index purchase arrest | Ref. | Ref. |
| Less than or equal to 2 years | 6.62 (6.19-7.09) | 5.81 (5.41-6.24) |
| More than 2 years | 3.91 (3.68-4.15) | 4.27 (4.00-4.55) |
| **Purchaser characteristics** |  |  |
| Race and ethnicity |  |  |
| American Indian |  | 2.10 (1.67-2.65) |
| Asian |  | 1.16 (1.04-1.30) |
| Black |  | 2.94 (2.68-3.23) |
| Hispanic |  | 1.59 (1.49-1.71) |
| Other |  | 1.38 (1.10-1.73) |
| Pacific Islander |  | 1.69 (1.32-2.16) |
| White |  | Ref. |
| **Purchaser community characteristics** |  |  |
| PC1^b^ |  | 1.11 (1.09-1.13) |
| PC2^b^ |  | 0.91 (0.88-0.94) |
| PC3^b^ |  | 0.93 (0.89-0.96) |
| RUCA |  |  |
| Metro |  | Ref. |
| Non-metro |  | 0.99 (0.91-1.08) |
| ICE-race^c^ |  | 0.75 (0.65-0.84) |
| Ratio of males to females aged 15 to 30 years |  | 0.92 (0.72-1.19) |

Results from robust regression models with robust standard errors.

^a^Includes rifle/shotgun combination firearms.

^b^Principle components were created from ICE-income, proportion renters, proportion single parent households with children, proportion with bachelor’s degree or higher, proportion unemployed, median household income ($), median home value, and proportion receiving welfare. Three principles components explained 75% of the variance.

^c^ICE-race ranges from −1 (all residents belong to the least privileged group) to +1 (all residents belong to the most privileged group), with least and most privileged groups classified as Black and White based on socially constructed hierarchies.

Table s5. Association between index purchase characteristics, prior criminal history (defined as an arrest within 2 years, an arrest 2+ years prior, and no prior arrests) and subsequent arrest for firearm violent crime

|  | **Model 1** | **Model 2** |
| --- | --- | --- |
|  | **OR (95% CI)** | **OR (95% CI)** |
| **Index purchase characteristics** |  |  |
| Gun show |  |  |
| Yes | 0.96 (0.79-1.17) | 1.02 (0.84-1.23) |
| No | Ref. | Ref. |
| Transaction type |  |  |
| Dealer’s sale | Ref. | Ref. |
| Non-roster peace officer | 0.29 (0.18-0.48) | 0.36 (0.21-0.60) |
| Other | 0.63 (0.51-0.79) | 0.73 (0.58-0.93) |
| Out of state registration | 1.00 (0.89-1.13) | 0.97 (0.80-1.18) |
| Pawn redemption | 1.46 (1.15-1.86) | 1.36 (1.05-1.76) |
| Private party transfer | 0.76 (0.70-0.83) | 0.83 (0.76-0.90) |
| Firearm type |  |  |
| Handgun | Ref. | Ref. |
| Rifle^a^ | 0.66 (0.35-1.25) | 0.71 (0.31-1.63) |
| Shotgun | 0.55 (0.28-1.08) | 0.73 (0.31-1.73) |
| Firearm category |  |  |
| Bolt action | 0.60 (0.49-0.74) | 0.63 (0.51-0.79) |
| Other/unknown | 1.07 (0.88-1.31) | 1.07 (0.87-1.31) |
| Pump action | 1.47 (1.17-1.85) | 1.12 (0.88-1.43) |
| Revolver | 0.90 (0.82-1.00) | 0.97 (0.87-1.08) |
| Semi-automatic | Ref. | Ref. |
| Caliber |  |  |
| Medium | 0.95 (0.85-1.07) | 0.87 (0.77-0.98) |
| Other | 1.14 (0.60-2.17) | 1.11 (0.48-2.57) |
| Small | Ref. | Ref. |
| Large | 0.91 (0.80-1.02) | 0.82 (0.73-0.94) |
| Low cost |  |  |
| Yes | 1.58 (1.39-1.79) | 1.13 (0.98-1.29) |
| No | Ref. | Ref. |
| Total number prior purchases | 1.00 (0.99-1.01) | 1.02 (1.01-1.03) |
| Age at index purchase |  | 0.94 (0.93-0.94) |
| **Criminal history** |  |  |
| Time from last pre-index purchase arrest to index purchase |  |  |
| No pre-index purchase arrest | Ref. | Ref. |
| Less than or equal to 2 years | 7.26 (6.74-7.81) | 6.24 (5.76-6.76) |
| More than 2 years | 4.18 (3.95-4.42) | 4.40 (4.14-4.67) |
| **Purchaser characteristics** |  |  |
| Race and ethnicity |  |  |
| American Indian |  | 2.09 (1.66-2.63) |
| Asian |  | 1.16 (1.03-1.30) |
| Black |  | 2.93 (2.67-3.22) |
| Hispanic |  | 1.59 (1.48-1.70) |
| Other |  | 1.38 (1.10-1.73) |
| Pacific Islander |  | 1.69 (1.33-2.15) |
| Unknown/missing |  | 1.01 (0.72-1.42) |
| White |  | Ref. |
| **Purchaser community characteristics** |  |  |
| PC1^b^ |  | 1.11 (1.09-1.13) |
| PC2^b^ |  | 0.91 (0.88-0.94) |
| PC3^b^ |  | 0.93 (0.90-0.97) |
| RUCA |  |  |
| Metro |  | Ref. |
| Non-metro |  | 0.99 (0.91-1.08) |
| ICE-race^c^ |  | 0.74 (0.65-0.84) |
| Ratio of males to females aged 15 to 30 years |  | 0.92 (0.72-1.19) |

Results from robust regression models with robust standard errors.

^a^Includes rifle/shotgun combination firearms.

^b^Principle components were created from ICE-income, proportion renters, proportion single parent households with children, proportion with bachelor’s degree or higher, proportion unemployed, median household income ($), median home value, and proportion receiving welfare. Three principles components explained 75% of the variance.

^c^ICE-race ranges from −1 (all residents belong to the least privileged group) to +1 (all residents belong to the most privileged group), with least and most privileged groups classified as Black and White based on socially constructed hierarchies.

Table s6. Interactions between index firearm purchase characteristics and prior criminal history (defined as an arrest within 2 years, an arrest 2+ years prior, and no prior arrests), and subsequent arrest for firearm violent crime in their association with subsequent arrest for firearm violent crime

|  | **Time from last pre-index purchase arrest to index purchase** | | |
| --- | --- | --- | --- |
|  | Among those with no arrest | Among those whose last pre-index purchase arrest was less than or equal to 2 years before index purchase | Among those whose last pre-index purchase arrest was more than 2 years before index purchase |
|  | OR (95% CI) | OR (95% CI) | OR (95% CI) |
| **Low cost** |  |  |  |
| Yes | 1.48 (1.23-1.79) | 0.93 (0.71, 1.23) | 0.90 (0.72, 1.12) |
| No | Ref. | Ref. | Ref. |
| **Transaction type** |  |  |  |
| Dealer's sale | Ref. | Ref. | Ref. |
| Non-roster peace officer | 0.43 (0.26-0.71) | --^a^ | 0.10 (0.01, 0.77) |
| Other | 0.63 (0.46-0.88) | 1.26 (0.62, 2.53) | 0.80 (0.55, 1.16) |
| Out of state registration | 0.86 (0.63-1.18) | 1.00 (0.74, 1.34) | 1.09 (0.82, 1.47) |
| Pawn redemption | 1.74 (1.21-2.50) | 1.00 (0.60, 1.68) | 1.25 (0.81, 1.92) |
| Private party transfer | 0.72 (0.63-0.83) | 1.06 (0.85, 1.32) | 0.89 (0.77, 1.02) |
| **Firearm type** |  |  |  |
| Handgun | Ref. | Ref. | Ref. |
| Rifle^b^ | 0.68 (0.29-1.58) | 0.95 (0.40, 2.25) | 0.69 (0.30, 1.60) |
| Shotgun | 0.69 (0.29-1.65) | 1.12 (0.45, 2.75) | 0.69 (0.29, 1.64) |
| **Caliber** |  |  |  |
| Medium | 0.91 (0.75-1.09) | 0.82 (0.61, 1.11) | 0.85 (0.70, 1.03) |
| Other | 1.14 (0.48-2.68) | 1.46 (0.59, 3.57) | 1.05 (0.44, 2.47) |
| Small | Ref. | Ref. | Ref. |
| Large | 0.88 (0.73-1.06) | 0.75 (0.55, 1.00) | 0.79 (0.65, 0.96) |

Results from conditional logistic regression models. All models control for gun show, transaction type, firearm type, caliber, cost, number of purchases, and purchaser demographic and community covariates as in Model 2, Table 2. Significant interactions at alpha 0.20 (likelihood ratio test) are presented.

^a^Estimate not presented due to sparse data.

^b^Includes rifle/shotgun combination firearms.

Table s7. Association between post-index purchase criminal history and subsequent arrest for firearm violent crime

|  | **Model 1** | **Model 2** |
| --- | --- | --- |
|  | **OR (95% CI)** | **OR (95% CI)** |
| Any post-index purchase arrest |  |  |
| No | Ref. | Ref. |
| Yes | 2.26 (2.10-2.44) | 1.92 (1.77-2.08) |

Results from conditional logistic regression models with robust standard errors. Includes the same set of variables as the primary models including pre-index purchase arrests (alternative definition), firearm characteristics, transaction characteristics and individual and community control variables.
